# Durability is improved by both low and high intensity endurance training

**DOI:** 10.3389/fphys.2023.1128111

**Published:** 2023-02-16

**Authors:** Pekka Matomäki, Olli J. Heinonen, Ari Nummela, Jari Laukkanen, Eero-Pekka Auvinen, Leena Pirkola, Heikki Kyröläinen

**Affiliations:** ^1^ Faculty of Sport and Health Sciences, University of Jyväskylä, Jyväskylä, Finland; ^2^ Paavo Nurmi Centre & Unit for Health and Physical Activity, University of Turku, Turku, Finland; ^3^ Finnish Institute of High Performance Sport KIHU, Jyväskylä, Finland; ^4^ Central Finland Healthcare District, Department of Medicine, Jyväskylä, Finland; ^5^ Department of Medicine, Institute of Clinical Medicine, University of Eastern Finland, Kuopio, Finland

**Keywords:** durability, low intensity training, high intensity training, perceived exertion, cardiovascular drift

## Abstract

**Introduction:** This is one of the first intervention studies to examine how low- (LIT) and high-intensity endurance training (HIT) affect durability, defined as ‘time of onset and magnitude of deterioration in physiological-profiling characteristics over time during prolonged exercise’.

**Methods:** Sedentary and recreationally active men (n = 16) and women (n = 19) completed either LIT (average weekly training time 6.8 ± 0.7 h) or HIT (1.6 ± 0.2 h) cycling for 10 weeks. Durability was analyzed before and after the training period from three factors during 3-h cycling at 48% of pretraining maximal oxygen uptake (VO_2max_): 1) by the magnitude and 2) onset of drifts (i.e. gradual change in energy expenditure, heart rate, rate of perceived exertion, ventilation, left ventricular ejection time, and stroke volume), 3) by the ‘physiological strain’, defined to be the absolute responses of heart rate and its variability, lactate, and rate of perceived exertion.

**Results:** When all three factors were averaged the durability was improved similarly (time x group *p* = 0.42) in both groups (LIT: *p* = 0.03, g = 0.49; HIT: *p* = 0.01, g = 0.62). In the LIT group, magnitude of average of drifts and their onset did not reach statistically significance level of *p* < 0.05 (magnitude: 7.7 ± 6.8% vs. 6.3 ± 6.0%, *p* = 0.09, g = 0.27; onset: 106 ± 57 min vs. 131 ± 59 min, *p* = 0.08, g = 0.58), while averaged physiological strain improved (*p* = 0.01, g = 0.60). In HIT, both magnitude and onset decreased (magnitude: 8.8 ± 7.9% vs. 5.4 ± 6.7%, *p* = 0.03, g = 0.49; onset: 108 ± 54 min vs. 137 ± 57 min, *p* = 0.03, g = 0.61), and physiological strain improved (*p* = 0.005, g = 0.78). VO_2max_ increased only after HIT (time x group *p* < 0.001, g = 1.51).

**Conclusion:** Durability improved similarly by both LIT and HIT based on reduced physiological drifts, their postponed onsets, and changes in physiological strain. Despite durability enhanced among untrained people, a 10-week intervention did not alter drifts and their onsets in a large amount, even though it attenuated physiological strain.

## 1 Introduction

Recently, the term *durability* has been defined as ‘time of onset and magnitude of deterioration in physiological-profiling characteristics during prolonged exercise’ (Maunder et al., 2021). Durability can be seen as a form of fatigue resistance. However, fatigue resistance is usually connected to neuromuscular fatigue during maximal and short performances. Durability, on the other hand, is usually linked to gradual fatiguing process in *prolonged* (i.e. several hours) *submaximal* exercise with usually physiological or psychological origin. Durability may be estimated directly by evaluating the magnitudes and onsets of *drifts*, defined as gradual changes in key physiological variables during prolonged submaximal exercise (Smyth et al., 2022). Durability may also be assessed by measuring a maximal performance capacity immediately after prolonged exercise (Maunder et al., 2021; Van Erp et al., 2021). Decreased absolute physiological responses, e.g. lactate and heart rate level, during submaximal exercise after a training period may also represent attenuated physiological responses and thus improved durability.

Many physiological drifts occur during prolonged low-intensity exercise, e.g. increased energy expenditure (EE) (Rønnestad et al., 2011; Hopker et al., 2017), which is coupled with upward drifts of ventilation (VE) (Ekelund, 1967; Martin et al., 1981; Hopker et al., 2017), rate of perceived exertion (RPE) (Rønnestad et al., 2011; Hopker et al., 2017; Wills et al., 2019), and heart rate (HR) (Ekelund, 1967; Rønnestad et al., 2011; Mullins et al., 2015). HR drift is the most widely used drift when studying durability (Maunder et al., 2021; Smyth et al., 2022), and it is also related to neuromuscular fatigue (Coyle and González-Alonso, 2001). Inversely, stroke volume (SV) (Ekelund, 1967) and linear heart rate variability (HRV) indices (Moreno et al., 2013) form downward drifts.

High durability, i.e., ability to resist physiological changes, has clear advantages. For example, a marathoner with high durability can resist the inevitable performance deteriorating changes and maintain performance capacity for prolonged periods of time. In line with this, lower HR drift in marathon running is associated with prolonged onset of drift and faster relative speed (Smyth et al., 2022) and greater HR drift is associated with decrease in running speed at the later stage of the race (Billat et al., 2020) as well as worse total time (Billat et al., 2020; Smyth et al., 2022). Larger HR drift predicts also decreased maximal oxygen uptake (VO_2max_) at fatigued state (Wingo et al., 2005). EE drift has been strongly associated with a decrease in 5 min maximum performance in fatigued state (Passfield and Doust, 2000). Lately, it has been demonstrated that power profile does not decline after exhausting exercise (1500–2000 kJ) as much in successful professional cyclists compared to lesser successful ones (Van Erp et al., 2021). In addition, durability is a required feature in military training, because physical and mental functionality of soldiers should be maintained at high levels also in a fatigued state (Rudzki, 1989; Wills et al., 2019).

The most effective training methods to improve durability are not known. In some studies, strenuous (Hurley et al., 1986; Coggan et al., 1993) and high-volume moderate-intensity (Phillips et al., 1996; Carter et al., 2001) endurance training has led to reduced physiological drifts during prolonged exercise among untrained individuals. Well-trained athletes have not increased durability with endurance training alone (Hausswirth et al., 2010; Rønnestad et al., 2011), although the amount of low-intensity training (LIT) may be linked to increased durability (Spragg et al., 2022). Concurrent strength and endurance training has had a positive effect among athletes (Rønnestad et al., 2011).

There are no reports on intervention studies focusing on durability. Therefore, we wanted to understand how much and in which methods durability can be improved. Our main aim was to study durability before and after high- (HIT) and low-intensity endurance training of 10 weeks in recreationally active participants.

## 2 Materials and methods

### 2.1 Study design and protocol

Based on VO_2_ drifts in interventions (Coggan et al., 1993; Carter et al., 2001) and our pilot studies, we estimated that intervention would cause an average 3 p. p. (percentage point) reduction in EE drift in the HIT group, and 6 p. p. reduction in the LIT group with 3 p. p. standard deviation. Based on these findings, power calculations give group sample size of 16 ( α=0.05;β=0.80 ).

**FIGURE 1:**
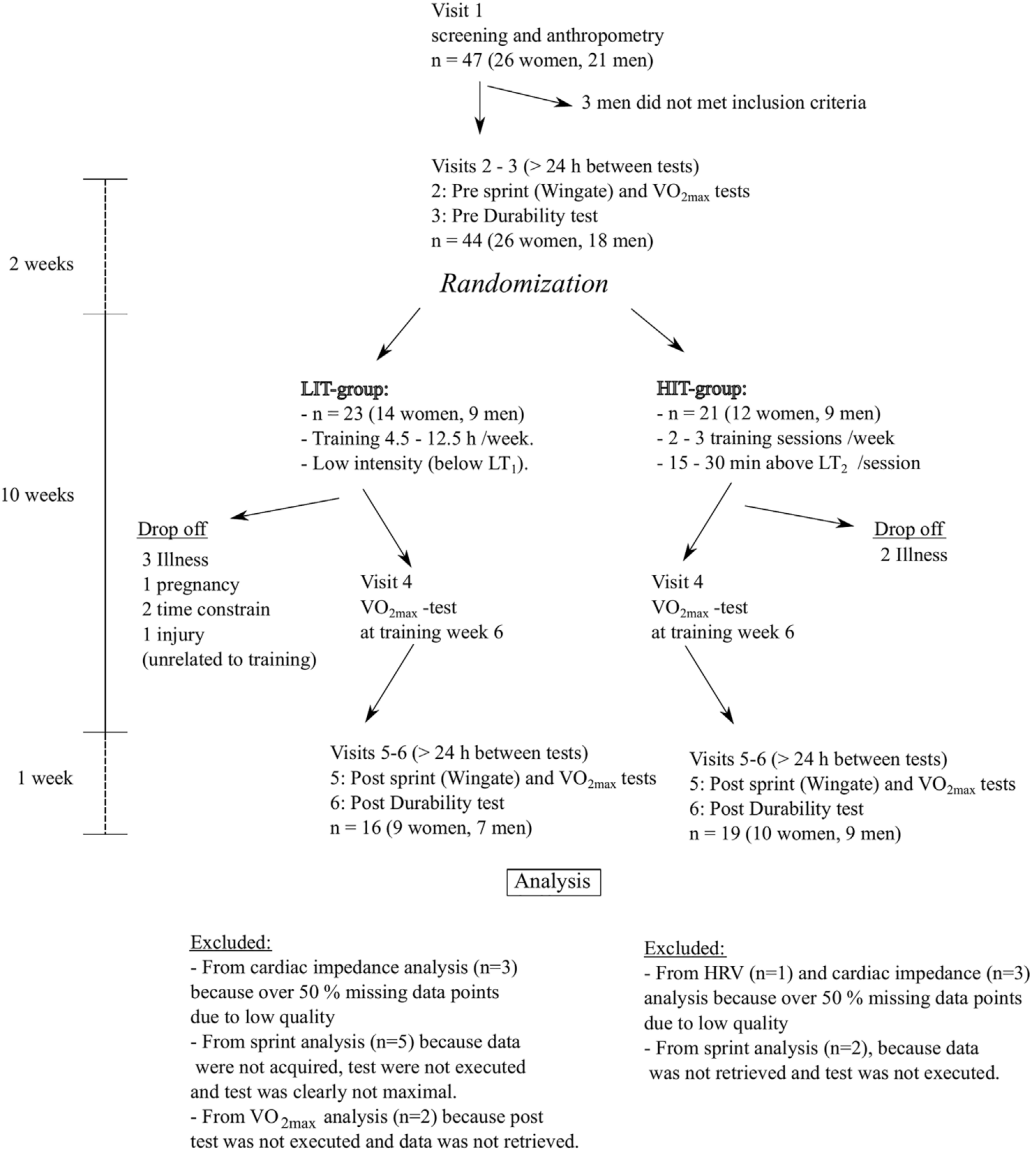
Flow chart and study design. Performance tests included VO_2max_ and sprint tests, and durability. *LIT* Low intensity training. *HIT* High intensity training. *HRV* heart rate variability. *VO*_*2max*_ maximal oxygen uptake. *LT*_*1*_ First lactate threshold. *LT*_*2*_ Second lactate threshold.

The study included 6 visits to the laboratory (Figure 1): three before the training intervention, one during intervention, and two after intervention. All subjects provided written informed consent, and the study was approved by the Ethical Committee of the Central Finland Healthcare District (8U/2020), complying with the Declaration of Helsinki.

### 2.2 Subjects

Healthy sedentary or recreationally active (endurance exercise less than 6 h/wk) adults aged 18–40 years were recruited through social and print media announcements. Additionally, participants were not included if they had hypertension, pregnancy, or were nursing, or they had been diagnosed an upper respiratory infection or other acute illnesses within 2-weeks before the first laboratory visit. A total of 47 subjects were invited to the screening visit which included assessments of body height, resting ECG, blood pressure, health status, and medication (Figure 1: flow chart). Body mass and fat free mass were measured by bioelectrical impedance (InBody 770, Biospace Ltd., Seoul, Korea) in the morning after at least 8 h fasting. Forty-four subjects met the inclusion criteria and started the training intervention. Finally, 35 subjects (basic characteristics in Table 1) finished the entire study.

**TABLE 1:** Basic characteristics (mean, SD) at the beginning of the study.

		Body mass (kg)	Height (cm)	Fat percent (%)	Age (y)	VO_2max_ (l/min)	P_max_ (W)	P_LT1_ (W)	P_LT2_ (W)	Sprinting fatigue ratio (%)
LIT	women (n = 9)	73.2 (15.2)	168 (6)	30.2 (6.9)	33 (5)	2.54 (0.12)	188 (9)	83 (5)	134 (8)	-3.0 (3.7) (n = 7)
	men (n = 7)	88.4 (9.7)	178 (4)	24.7 (7.9)	34 (6)	3.62 (0.14)	272 (9)	121 (9)	204 (9)	-1.4 (6.0) (n = 4)
HIT	women (n = 10)	62.8 (9.0)	164 (7)	27.6 (5.9)	30 (5)	2.33 (0.04)	176 (3.4)	79 (4)	128 (4)	-0.1 (3.0) (n = 9)
	men (n = 9)	87.7 (9.8)	180 (7)	22.3 (6.0)	34 (5)	3.25 (0.13)	251 (14)	115 (10)	191 (13)	-1.5 (7.9) (n = 8)

*LIT* low intensity training group; *HIT* High Intensity Training group. *VO*_*2max*_ Maximal oxygen uptake. *P*_*max*_ Maximum aerobic power. *P*_*LT1*_ Power at the first lactate threshold. *P*_*LT2*_ Power at the second lactate threshold. *Sprinting fatigue ratio* Ratio of (15 s sprint after durability test - 15 s sprint rest)/(15 s sprint rest).

### 2.3 Laboratory tests and analyses

*Visit 2, 4, & 5: The sprint (Wingate) and VO*_*2max*_ were performed individually at the same time of the day (±2 h). Visit 4 included only the VO_2max_-test. The subjects were advised to refrain from caffeine and alcohol 24 h before the tests and eating 3 h prior. Before each performance test, body mass was recorded (seca 719, seca GmbH & Co. KG., Hamburg, Germany) with cycling clothes on and shoes removed, then 300 g were subtracted as the weight of clothes.

15 s *Wingate test (sprinting ability)* was used to measure a fatigue of the neuromuscular system after a durability test. A 10-min warming up at 50 W followed by 10 s countdown phase to reach maximum pedaling rate with the load (7.5% of body weight) followed by 15 s all out maximal cycling (Monark 894E, Monark Exercise AB, Sweden). Subjects were verbally encouraged by researchers. Subjects were familiarized with the Wingate test on a separate day before the test.

*Incremental VO*_*2max*_ *cycling test.* After the Wingate test, subjects remained sitting for 10 min, followed by a 25-min active recovery (walking around or 50 W cycling). Exactly 35 min after termination of the Wingate test, a step-incremental cycling test (Monark LC4, Monark Exercise AB, Sweden) was initiated. Initial power was 40 W for women and 50 W for men with increments of 20—25 W (women) and 30 W (men) at 3-min intervals. Subjects were verbally encouraged during the last stages by researchers. The ergometer (Monark LC4) was calibrated at the beginning of the study. Calibration was checked weekly with a 4000 g weight, following instructions by the manufacturer. Gas exchange was measured breath-by-breath (Jaeger Vyntus TM CPX, CareFusion Germany 234 GmbH, Hoechberg, Germany) and HR was monitored with a Garmin Forerunner 945 (Garmin Ltd., Taiwan). Means from the last minute of every stage were used in analysis. In the last minute of each state, blood lactate was measured by fingertip sampling (EKF-diagnostic GmbH Ebendorfer Chaussee 3, Germany) and RPE (Borg Scale 0–10) was recorded. VO_2max_ was defined as the highest continuous 60 s mean oxygen consumption. Maximal aerobic power (P_max_) was defined as the weighted mean of the last 3 min of the test: power of last completed stage (W) + [time (s) of unfinished state]/(180 s) × increment (W). The first lactate threshold (LT_1_) was defined as the lowest value of the lactate/VO_2_ -ratio, and the second lactate threshold (LT_2_) as a sudden and sustained increase in blood lactate concentration (Faude et al., 2009). Two researchers independently determined the thresholds. In case of disagreement, a third opinion was obtained.

*Durability* is defined as ‘time of onset and magnitude of deterioration in physiological-profiling characteristics over time during prolonged exercise’ (Maunder et al., 2021). In this study, the original definition of durability was interpreted through three different factors:

1) The *magnitude of physiological drifts* (defined as a gradual change in physiological variables during exercise): Change in EE, HR, RPE, VE, imputed left ventricular ejection time (LVET), and imputed stroke volume (SV) between 30 min and 180 min in the durability test.
2) The *time of onset* of a drift, which was defined to be the time when the value of the drift has risen (or fallen) a predetermined amount. The thresholds were arbitrarily chosen, as in (Smyth et al., 2022), and they were +5% (HR, VE), +2.5% (EE), +2 steps (RPE), and -1.5% (imputed LVET).
3) *The physiological strain*, which was defined as the absolute levels of HR, HRV, RPE, and blood lactate from the durability test. The effect of prolonged exercise on sprinting performance was done by inspecting the sprinting fatigue ratio: Δ sprint = (sprint_fatigue_—sprint_rest_)/sprint_rest_, where sprint_fatigue_ was the mean of 15-s Wingate power after the durability test and sprint_rest_ at rested state.

To get a wider overall picture, durability was not understood as a single variable, but rather as a phenomenon which is illustrated by all these three different factors together.

*Visit 3 and 6: Durability tests* were performed individually for each subject at the same time of the day (±2 h). The subjects were advised to have a standard meal 2.5–3 h before entering the laboratory. They were instructed to document the timing and contents of the meal, and to repeat it before the second durability test. Before the durability test, body mass was measured (Seca 719), then followed by a rest period on bench while being prepared for the non-invasive impedance cardiography (Physioflow, 2016) (PhysioFlow PF-07 Enduro, Manatec Biomedical, France) with PF50 PhysioFlow electrodes to measure SV and LVET.

Thereafter, the subjects cycled for 3 h (Monark LC4) with a predetermined power, 50% VO_2max_, measured on incremental test, but not more than 95% LT_1_ to ensure that each participant cycled at low intensity zone. In pre-test the realization was 48 ± 4% VO_2max_ and 87 ± 8% LT_1_. The same absolute power in pre- and post-tests was used. Ten-minute measurement slots were repeated every 30 min. During the first 20 min, the subjects chose their preferred position and cadence (over 60 rpm), which were recorded and maintained during the subsequent 10-min measurement slots in both pre- and post-tests. Between the 10-min measurement slots, subjects could adjust position and cadence (>60 rpm) freely. Gas exchange was measured breath-by-breath during each 10-min measurement slots (Jaeger Vyntus TM CPX), after which RPE (0–10) was recorded. Blood was drawn from fingertips at 0, 1, 2, and 3 h for analyzing blood lactate. The 15-s Wingate test was performed within the first minute after finishing the 3 h test.

Hydration (0.3% NaCl solution) was available *ad libitum*. Water intake during 3 h tests was measured and in the LIT group, it was 1.2 (0.4) l and 1.1 (0.4) l in pre- and post-tests, respectively. In the HIT group, the respective values were 1.3 (0.4) l and 1.1 (0.4) l. During the test, carbohydrates were given as a 2:1:1 mixture containing maltodextrin, fructose, and glucose dissolved into 1 dl of 0.3% NaCl. Carbohydrate intake was individualized, and the amount was related to the cycling power, calculated to cover 50% of theoretical energy expenditure where 18% gross efficiency was assumed (Ettema and Loras, 2009). A maximum of 75 g of carbohydrates were given per hour to minimize gastrointestinal problems (Jeukendrup, 2014). The carbohydrate intake was identical in pre- and post-tests (mean ± SD: 51 ± 14 g/h).

*Energy expenditure (EE)* was calculated using equation $EE\;\left(kJ/\min\right)=\left(5.05\times RER+16.1\right)\times {VO}_{2}\left(l/\min\right)$ (Keskinen et al., 2004), where RER is respiratory exchange ratio.

*Heart rate variability (HRV), cardiac impedance, and multiple imputations.* In the HRV analysis, low frequency (0.04–0.15 Hz) and high frequency (0.15–1.1 Hz) bands were used. The higher than usual frequency limit of 1.1 Hz was chosen to include the respiratory frequency during exercise. HR and HRV data were collected with Garmin HRM-Pro or HRM-dual belt (Garmin Ltd., Taiwan), with 1000 Hz resolution frequency, and analyzed with Kubios HRV Premium (Version 3.5.0, Kubios Oy, Kuopio, Finland). Medium automatic quality detection with a 5% acceptance threshold and an automatic beat correction method were used. Low- and high-frequency spectrums were calculated applying Lomb-Scargle periodogram with 0.02 Hz smoothing window. The HRV samples were 8-min subintervals within 10-min measurement intervals of the durability test. Only data points with effective data length at least 95% were included in the final analysis.

In the analysis, after removing all subjects with more than 50% missing data points (six from cardiac impedance measurements and one from HRV), 7.0% of HRV and 13.2% of cardiac impedance data were missing. Multiple imputations were used to fill in missing data. Drifts in imputed LVET and SV were interpolated from regression lines.

### 2.4 LIT and HIT training

Subjects were randomized into two training groups, applying minimization method (Hu and Hu, 2012) after the first three visits to laboratory. Training zones were determined based on lactate thresholds. The subjects were instructed to continue their daily physical activities (commuting, non-physical hobbies, etc.), but all strenuous exercise in addition to LIT or HIT was not allowed. The exact training programs are given in Supplementary Material 1.

LIT consisted of cycling under LT_1_-power. The weekly 5–6 training days included long (1.5–4 h), medium (1–1.5 h), and short (45–60 min) exercises. Weekly training hours progressed individually (see Progression-paragraph below) based on perceived exertion from 4.5 up to maximum 12.5 h. Subjects did their training mostly outdoors with their own bicycles with Rally RK200 dual-sensing power meters (Garmin Ltd., Taiwan). A possibility for indoors cycling with trainer or Wattbike Trainer (Wattbike Ltd., Nottingham, UK) was given. Three (out from 16) subjects did their training completely indoors, and the others did 3% of their training indoors.

HIT consisted of 2—3 weekly indoor training sessions with Wattbike Trainers, or indoor trainer with their own bicycles with Rally RK200 dual-sensing power meters. Training consisted of high-intensity work intervals 3–7 min long with recovery periods ¾ of the work interval duration. In the first training week, there were 15 min of cumulative high-intensity time in a training session, and it progressed individually (see Progression-paragraph below) up to 30 min per session. Each session included 10 min warm up and cool down. These, as well as recovery periods, were done with power <60 W, and high-intensity segments were initially 110% LT_2_ power ( ±15 W).

In both groups, RPE (0–10) was reported from each training session. HR from each session was recorded with the Garmin HRM-Pro heart belt. In the LIT group, power data were recorded with power meters (Rally RK200), and in the HIT group, power data were collected from the Wattbike Trainer or power meters (Rally RK200). All data was transferred after the session to AthleteMonitoring app (AthleteMonitoring, FITSTATS Technologies, Inc., Moncton, Canada), from which training realization was actively monitored weekly by the first author. In the HIT group, if all weekly training had RPE ≤ 6 and HR did not rise above LT_2_-threshold, the power was increased by 10%. In the LIT group, the subjects were actively given feedback whether training was at the prescribed level. Apart from the first HIT-session, all sessions were unsupervised. Training power was modified in both groups according to VO_2max_ -test (visit 4) at training week 6.

Daily physical activity was gathered by measuring heart rate from wrist continuously by Garmin Forerunner 945 for two to 4 weeks before the start of the intervention. This was done to estimate the baseline endurance training before training intervention.

*Training zones and load.* Training was monitored by distributing cycling power output to five zones (Cejuela-Anta and Esteve-Lanao, 2011): Z1 (below LT_1_—10 W); Z2 (LT_1_—10 W to LT_1_ + 10 W); Z3 (LT_1_ + 10 W to LT_2_—10 W); Z4 (LT_2_—10 W to LT_2_ + 10 W); Z5 (above LT_2_ + 10 W). For each zone, a weighting factor was linked (in an ascending order: 1, 2, 3, 4, 7.5) and training load was calculated by multiplying the factor by the time spent in the zone (Cejuela-Anta and Esteve-Lanao, 2011).

Targeted power of LIT was designed to be at Z1 and Z2, and HIT at Z4 and Z5. Heart rate was divided into three zones (HR Zones 1—3) separated by LT_1_ and LT_2_. In addition, for the LIT group, it was calculated how many times their 30 s -average power exceeded Z2 -zone. For baseline physical activity before the start of the training intervention, Z0 was defined to be a zone below halfway between resting HR and LT_1_, and Z1b above Z0 and below LT_1_. During physical activity heart rate measurement part of the data was dismissed, 12 ± 15% of the gathered data, because of heart rate was not adequately recorded from wrist.

**FIGURE 2:**
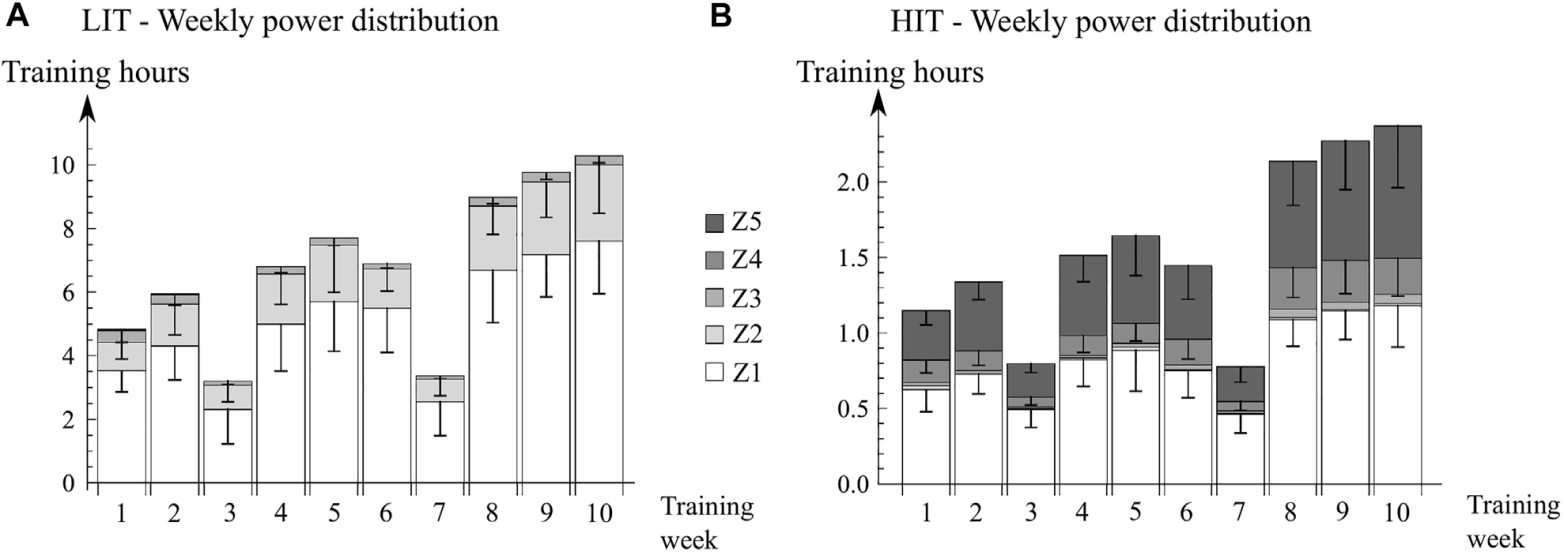
Mean weekly training power distribution for **(A)** LIT, SD for zones Z1, Z2, Z3; **(B)** HIT, SD for zones Z1, Z4, and Z5. Observe the different y-axis in figures **(A)** and **(B)**. The small reduction in hours in week 6 was caused by replacing one session by VO_2max_-test. Mean weekly training hours in the LIT group was 4.4 (range 4.0—4.8) times than that of the HIT group (excluding load reduction weeks). *LIT* Low intensity training. *HIT* High intensity training. *Power zones* Z1 (below LT_1_—10 W); Z2 (between LT_1_—10 W to LT_1_ + 10 W); Z3 (between LT_1_ + 10 W to LT_2_—10 W); Z4 (between LT_2_—10 W to LT_2_ + 10 W); Z5 (above LT_2_ + 10 W). *LT*_*1*_ First lactate threshold. *LT*_*2*_ Second lactate threshold.

*Training progression.* Both groups had 10 training weeks. Weeks 3 and 7 were load reduction weeks for enhancing recovery. Subjects’ training progression was linked to the perceived exertion. After each training week (excluding load reduction weeks), subjects were asked ‘How much the training has strained your week on a scale of 0–10?’, and the training time was increased more with lower exertion (see Supplementary Digital Content 1). For those finishing the study, adherence rate in training sessions was 98.4%. The weekly training intensity distribution is shown in Figure 2 and training realization in Table 2.

**TABLE 2:** Training realization, mean (SD), in LIT and HIT groups during 10- week training. Before training intervention there were no differences between the groups in HR distribution (*p* > 0.28).

	LIT (n = 16)	HIT (n = 19)
Before training		
Monitored days (d)	28 (12)	22 (13) (n = 17)
Physical activity (min/vrk)		
HR intensity distribution	171 (193)/1128 (184)/107 (89)/6 (7)/0.5 (0.4)	169 (203)/1091 (242)/108 (76)/6 (8)/0.4 (0.2)
Dismissed/Zone 0/Zone 1b/Zone 2/Zone 3		(n = 17)
Training charateristics		
Total training time (h)	68.2 (7.3)	15.6 (1.8)
Mean training volume (h/week)	6.8 (0.7)	1.6 (0.2)
Mean training frequency/week	4.8 (0.2)	2.4 (0.1)
Mean HR (% HR_max_)	63.4 (4.3)	76.1 (4.3)
Mean training session RPE	2.8 (1.4)	7.2 (1.9)
HR Zone 1 (%)/Power Zones Z1 & Z2 (%)	87.6 (17.5)/96.6 (2.5)	36.2 (10.1)/54.1 (4.7)
HR Zone 2 (%)/Power Zone Z3 (%)	12.3 (17.4)/3.2 (2.4)	37.7 (8.2)/1.9 (2.4)
HR Zone 3 (%)/Power Zones Z4 & Z5 (%)	0.1 (0.2)/0.2 (0.3)	26.1 (10.8)/44.0 (5.4)
30 s intervals > Z2 power/week	5.5 (7.8)	

*LIT* low intensity training group; *HIT* High intensity training group; *HR* Heart rate; *HR*_*max*_ Maximum heart rate; *RPE* Rate of perceived exertion. *Power Zones* Z1 (below LT_1_—10 W); Z2 (LT_1_—10 W to LT_1_ + 10 W); Z3 (LT_1_ + 10 W to LT_2_—10 W); Z4 (LT_2_—10 W to LT_2_ + 10 W); Z5 (above LT_2_ + 10 W). *LT*_*1*_ First lactate threshold. *LT*_*2*_ Second lactate threshold. *HR Training Zones* Z1 (below LT_1_); Z2 (between LT_1_ and LT_2_); Z3 (above LT_2_). For physical activity measurement *Dismissed* (HR data not acquired); *Z0* (below halfway between resting HR and LT_1_); *Z1b* (above Z0, below LT_1_).

### 2.5 Statistical analyses

Data are presented as mean (standard deviation). Statistical tests were calculated by SPSS 26.0 (SPSS Inc, Chicago, IL, United States) and Mathematica 13.0 (Wolfram Research, United States). Missing data on heart rate variability and cardiac impedance were handled by multiple imputation. To be conservative, 50 imputations were used (Licht, 2010). Pooled *p*-values were calculated applying z-method previously suggested (Licht, 2010).

Before performing the final analysis, it was determined if the magnitude of change in variables differed between the sexes (Kruskal-Wallis test) in 3 h test. Of all drifts and their onsets and physiological strains (88 tests), the only differences were detected in the RPE onset (*p* = 0.03) and lactate at 60 min time point (*p* = 0.04) in the HIT. As they were the only exceptions, it was decided to analyze females and males in a combined group. The Shapiro-Wilk test was used to examine normality.

If results were not normally distributed, group size was small (n < 10), or values were categorial (i.e. RPE, onset of drifts), the non-parametric Wilcoxon signed-rank-test was used for comparison between time points, and Mann-Whitney U-test for between-group comparison. When normality assumptions failed, but covariance matrices were homogenous by Box’s test, Pillai’s trace was utilized in ANOVA. If covariance assumption was not met, Kruskal-Wallis test was used. To calculate the average drifts and their onsets their weighted mean was calculated. In physiological strain, HRV responses were first averaged as a single variable. A harmonic mean *p*-value technique was applied to estimate the combined change in durability from all examined factors (drifts, their onset, and physiological strain) given all parameters weights proportional to their sample size. Post hoc correlations were done using Spearman correlations with RPE and Pearson correlations otherwise.

The effect size (ES) of differences for the main variables were calculated with a corrected effect size Hedge’s g, for which a non-central 95% confidence interval (CI) was calculated (Goulet-Pelletier and Cousineau, 2018). Effect size for between-group differences was calculated by subtracting within-group effect sizes from each other (Morris and DeShon, 2002). After non-parametric tests, effect size was calculated by a formula $Z/\sqrt{n}$ , where Z is the z-score, and n is the total number of subjects on which Z is based. As this corresponds to point biserial r, an analytical conversion is made to represent it as d-family ES (McGrath and Meyer, 2006):

$$\frac{r}{\sqrt{\left(1-r^{2}\right)\frac{n_{1}n_{2}}{{\left(n_{1}+n_{2}\right)}^{2}}}},$$

where $n_{1}$ and $n_{2}$ are the sizes of groups 1 and 2. A common language effect size (CLES) is also provided for main variables. When normality assumptions were met, a continuous method was used. In other cases, a sign-test (within-group CLES) or Mann-Whitney U-test (between-group CLES) were utilized (Caldwell and Vigotsky, 2020). The interpretation of CLES of X% is ‘the probability of a randomly selected individual’s variable increasing/being greater than the one from randomly selected individuals from the other group after the training is X%.’ Small, moderate, large, and very large effect size magnitudes for Hedge’s g were categorized as 0.20, 0.50, 0.80, and 1.2, and 56%, 64%, 71%, and 80% for CLES, respectively.

In figures, the statistical values of the durability test were pooled: *p*-values were changed to z-scores which were averaged, similar to the methodology of multiple imputation (Licht, 2010). Effect sizes were pooled following the suggestion by (Rosenthal and DiMatteo, 2001): Cohen d_i_ ES → Biseral r_i_ ES → Fisher $Z_{ri}\to$ Taking average ${\bar{Z}}_{r}=Mean\left(Z_{ri}\right)$ → Averaged biseral $\bar{r}$ ES → Averaged $\bar{d}$ ES → Averaged Hedge’s $\bar{g}$ , where *i* is the different time points from which averages were taken. Correlations were averaged using the same procedure. Common language effect sizes were averaged in continuous cases by averaging all ${µ}_{D}/\sigma_{D}$ values, where $\mu_{D}$ is the mean value of the observed difference and $\sigma_{D}$ its standard deviation. In non-normal cases, CLES was averaged by taking the average over all CLES’.

## 3 Results

*All factors combined*. When magnitude and onset of drifts and physiological strain were all averaged together, durability was improved in both LIT and HIT groups (LIT: combined harmonic *p* = 0.03, averaged Hedge’s g ES = 0.49, CLES = 68%; HIT: *p* = 0.01, ES = 0.62, CLES = 70%) with no difference between groups (time x group combined harmonic *p* = 0.42).

*Magnitudes of drifts.* There was no time x group difference in magnitude of drifts (combined harmonic *p* = 0.28). The averaged drifts from pre-to post-tests decreased (Table 3) in HIT (8.8 ± 7.9% vs. 5.4 ± 6.7%, combined harmonic *p* = 0.03, averaged Hedge’s g ES = 0.49, CLES = 65%), but did not reach statistically significance level of *p* < 0.05 in LIT (7.7 ± 6.8% vs. 6.3 ± 6.0%, *p* = 0.09, ES = 0.27, CLES = 62%).

**TABLE 3:** Mean (SD) magnitude of the drifts of physiological variables during 3 h durability test. Drifts of the imputed LVET and SV are extrapolated from regression lines.

		LIT	HIT	Between group change
				Time x group
EE drift (%)	Pre	**6.4 (4.8)**	**6.5 (6.8)**	
	Post	**4.0 (5.6)**	**3.0 (6.7)**	
	Change (p.p.)	**-2.4 (4.0)** *p* = 0.03	**-3.5 (6.4)** *p* = 0.03	*p* = 0.90
		ES = -0.45 (-0.89—-0.08)	ES = -0.51 (-1.00—-0.08)	ES = 0.06 (-0.62—0.76)
HR drift (bpm)	Pre	**15.1 (7.0)**	**16.7 (8.7)**	
	Post	**13.2 (7.3)**	**11.7 (6.9)**	
	Change (bpm)	**- 1.9 (5.4)** *p* = 0.18	**-4.9 (7.8)** *p* = 0.01	*p* = 0.19
		ES = -0.25 (-0.66–0.11)	ES = -0.62 (-1.13—-0.18)	ES = 0.36 (-0.31—1.08)
RPE drift	Pre	**3.3 (1.7)**	**3.5 (2.0)**	
	Post	**2.6 (1.9)**	**3.1 (1.3)**	
	Change	**-0.7 (1.6)** *p* = 0.10	**-0.4 (1.4)** *p* = 0.18	*p* = 0.26
		ES = -0.60 (-1.39–0.09)	ES = -0.44 (-1.10–0.22)	ES = 0.16 (-0.52—0.86)
VE drift (l/min)	Pre	**3.9 (2.9)**	**4.8 (4.8)**	
	Post	**2.9 (2.7)**	**2.3 (3.3)**	
	Change (l/min)	**-0.9 (2.2)** *p* = 0.11	**-2.5 (5.1)** *p* = 0.05	*p* = 0.39
		ES = -0.32 (-0.75–0.06)	ES = -0.59 (-1.2–0.04)	ES = 0.27 (-0.41—0.98)
Imputed LVET drift (ms)	Pre	**-8.3 (17.9) (n = 13)**	**-16.4 (11.7) (n = 16)**	
	Post	**-12.3 (16.3) (n = 13)**	**-8.8 (10.0) (n = 16)**	
	Change (ms)	**-4.0 (19.2)** *p* = 0.48	**+7.5 (15.7)** *p* = 0.10	*p* = 0.13
		ES = -0.23 (-0.89—0.39)	ES = 0.67 (-0.03—1.48)	ES = 0.9 (0.17—1.78)
Imputed SV drift (ml)	Pre	**-6.8 (9.1) (n = 13)**	**-3.2 (7.8) (n = 16)**	
	Post	**-5.7 (7.9) (n = 13)**	**-2.6 (5.7) (n = 16)**	
	Change (ml)	**+1.0 (11.1)** *p* = 0.75	**+0.6 (8.1)** *p* = 0.82	*p* = 0.72
		ES = 0.12 (-0.62—0.88)	ES = 0.08 (-0.51—0.68)	ES = 0.04 (-0.72—0.81)

*LIT* Low-intensity training group; *HIT* High-intensity training group; *EE* Energy expenditure; *HR* Heart rate; *RPE* Rate of perceived exertion; *VE* Ventilation; *LVET* Left ventricular ejection time; *SV* Stroke volume. *p. p*. percentage point. *Change* Absolute change between pre- and post-tests. Bold values are description values of the variables, *p p*-value. *ES* Hedge’s g effect size (95% confidence interval).

*Onset of drifts.* There were no time x group difference onset of drifts (combined harmonic *p* = 0.44). The average onset of drifts from pre-to post-tests prolonged (Table 4) in HIT (108 ± 54 min vs. 137 ± 57 min, combined harmonic *p* = 0.03, averaged Hedge’s g ES = 0.61, CLES = 63%), but did not reach statistically significance level of *p* < 0.05 in LIT (106 ± 57 min vs. 131 ± 59 min, *p* = 0.08, ES = 0.58, CLES = 68%).

**TABLE 4:** Mean (SD) time of onset of the physiological drifts: EE (2.5% increase), HR (5% increase), RPE (2 steps), VE (5% increase), LVET (1.5% decrease).

		LIT	HIT	Between group change
				Time x group
Time of onset EE increase of 2.5% (min)	Pre	**98 (59)**	**111 (60)**	
	Post	**126 (69)**	**161 (65)**	
	Change (min)	**+28 (76)** *p* = 0.14	**+50 (63)** *p* = 0.007	*p* = 0.62
		ES = 0.53 (-0.05—1.19)	ES = 0.96 (0.49—1.54)	ES = 0.43 (-0.24—1.16)
Time of onset HR increase of 5% (min)	Pre	**96 (50)**	**101 (52)**	
	Post	**118 (55)**	**115 (59)**	
	Change (min)	**+23 (66)** *p* = 0.17	**+14 (59)** *p* = 0.19	*p* = 0.56
		ES = 0.49 (-0.12—1.18)	ES = 0.43 (-0.05—0.96)	ES = 0.06 (-0.62—0.76)
Time of onset of RPE increase of 2 steps (min)	Pre	**128 (52)**	**120 (50)**	
	Post	**153 (46)**	**129 (45)**	
	Change (min)	**+26 (51)** *p* = 0.07	**+9 (50)** *p* = 0.42	*p* = 0.30
		ES = 0.66 (0.15—1.26)	ES = 0.26 (-0.22—0.77)	ES = 0.40 (-0.27—1.13)
Time of onset of VE increase of 5% (min)	Pre	**101 (56)**	**101 (59)**	
	Post	**122 (58)**	**134 (62)**	
	Change (min)	**+21 (32)** *p* = 0.03	**+33 (76)** *p* = 0.07	*p* = 0.72
		ES = 0.75 (0.45—1.16)	ES = 0.60 (0.03—1.24)	ES = 0.15 (-0.53—0.86)
Time of onset of Imputed LVET decrease of 1.5 % (min)	Pre	**110 (66) (n = 13)**	**106 (43) (n = 16)**	
	Post	**136 (63) (n = 13)**	**147 (49) (n = 16)**	
	Change (min)	**+26 (70)** *p* = 0.22	**+42 (74)** *p* = 0.04	*p* = 0.28
		ES = 0.48 (-0.11—1.16)	ES = 0.76 (0.19—1.44)	ES = 0.28 (-0.39—1.00)

*LIT* low intensity training group; *HIT* High intensity training group; *EE* Energy expenditure; *HR* Heart rate; *RPE* Rate of perceived exertion; *VE* Ventilation; *LVET* Left ventricular ejection time. *Change* Absolute change between pre- and post-tests. Bold values are description values of the variables, *p p*-value. *ES* Hedge’s g effect size (95% confidence interval).

**FIGURE 3:**
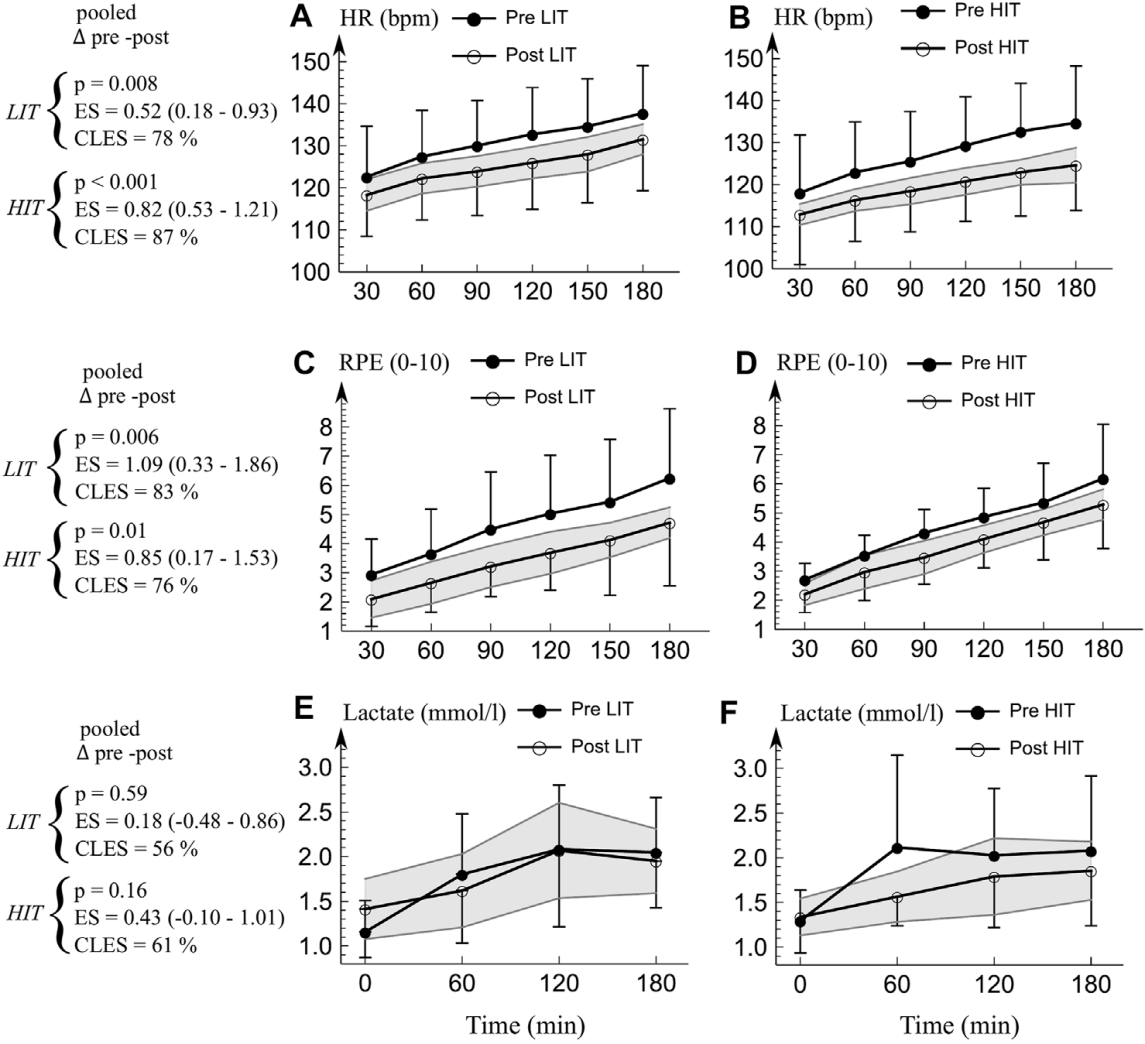
**(A–B)** Mean (SD) heart rate, **(C–D)** RPE, and **(E–F)** blood lactate concentration during durability test. Pooled *p*-values, ES (95% CI), and CLES were averaged from the time points of 30–180 min (60–180 min with lactate) to represent the average change. The gray area represents 95% confidence interval for the change in variable (i.e. if pre-value is outside gray area, the change in that time point is significant at *p* = 0.05 level). There were no time x group differences on average between the groups in HR, RPE, or lactate (*p* > 0.25). *LIT* Low intensity training group, *HIT* High intensity training group. *HR* Heart rate. *RPE* Rate of perceived exertion. *P p*-value. *ES* Effect size (Hedge’s g). *CLES* Common language effect size.

**FIGURE 4:**
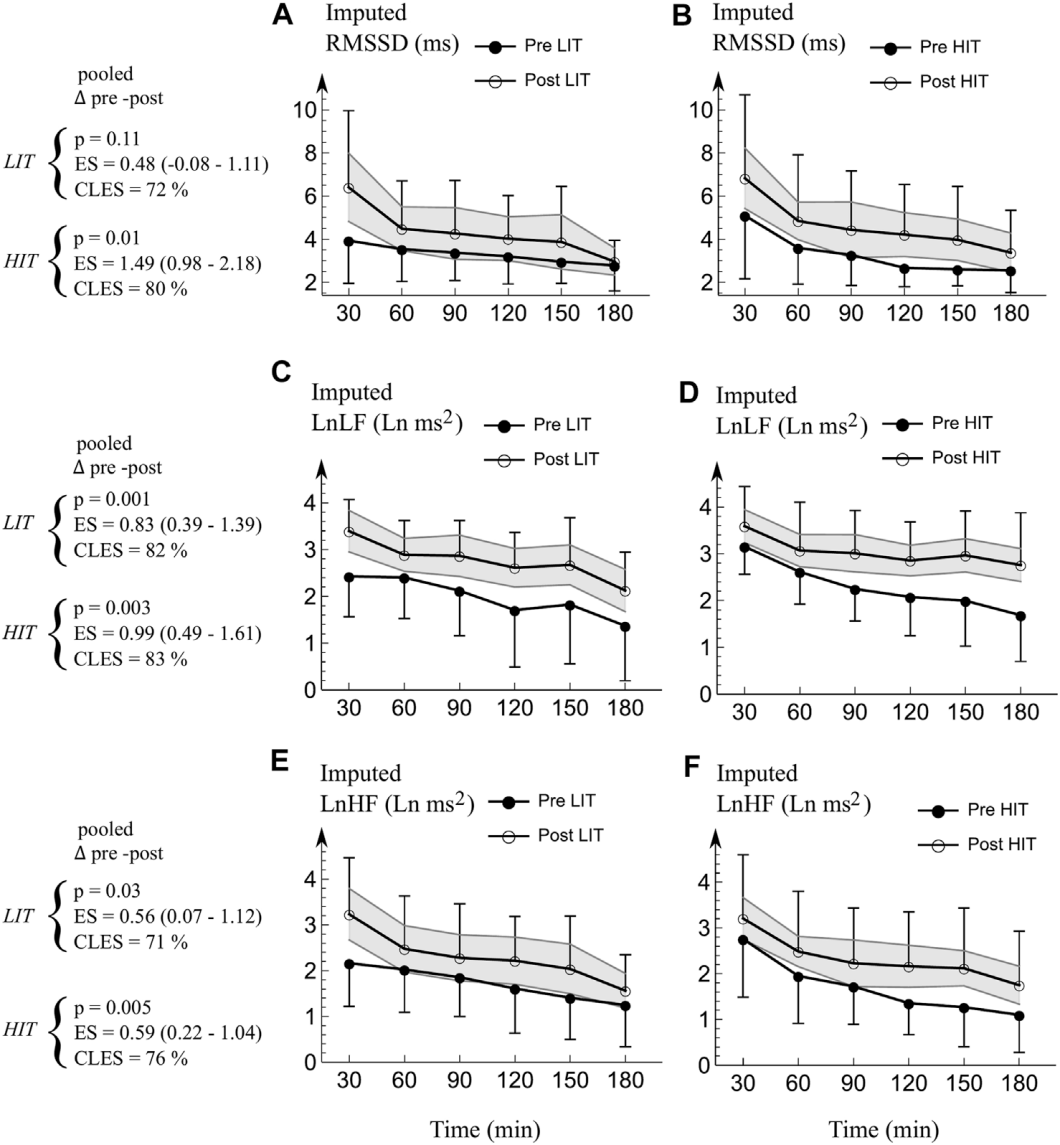
Mean (SD) imputed HRV responses to 3 h durability test. **(A,B)** RMSSD. **(C,D)** LnLF. **(E,F)** LnHF. Pooled *p*-values, ES (95% CI), and CLES were averaged from the time points of 30—180 min to represent the average change. The gray area represents 95% confidence interval for the change in variable (i.e. if pre-value is outside gray area, the change in that time point is significant at *p* = 0.05 level). ANOVA revealed no time x group differences (*p* > 0.10). *LIT* Low Intensity training group, *HIT* High Intensity training group. *HR* Heart rate. *RMSSD* Root mean square of successive RR interval differences. *LnLF* Natural logarithm of absolute power of the low-frequency band (0.04–0.15 Hz). *LnHF* Natural logarithm of absolute power of the high-frequency band (0.15–1.1 Hz). *p p*-value. *ES* Effect size (Hedge’s g). *CLES* Common language effect size.

*Physiological strain.* The averaged change in physiological strain (Figure 3; Figure 4) was large both in LIT (*p* = 0.01, ES = 0.60, CLES = 73%) and HIT (*p* = 0.005, ES = 0.78, CLES = 76%) The responses were similar on both groups (harmonic time x group *p* = 0.73).

*VO*_*2max*_ *and sprinting fatigue ratio.* There was a large time x group difference [*p* < 0.001, ES = 1.51 (0.80—2.43), CLES = 83%] in VO_2max_(l/min), which improved only in HIT (Table 5) [LIT: n = 14, Δ VO_2max_ = +0.02 (0.24) l/min, *p* = 0.71, ES = 0.04 (-0.15—0.24), CLES = 54%; HIT: Δ VO_2max_ = +0.32 (0.21) l/min, *p* < 0.001, ES = 1.55 (1.22—2.05); CLES = 100%].

**TABLE 5:** Basic physiological characteristics before and after the training (mean, SD).

		VO_2max_(I/min)	P_max_(W)	PLT_1 (W)	PLT_2 (W)	Sprinting fatigue ratio(%)
LIT n = 16	Pre	2.90 (0.62) (n = 14)	217 (47)(n = 14)	98 (28) (n = 15)	162 (42) (n = 15)	-2.4 (4.4) (n = 11)
	Post	2.93 (0.51) (n = 14)	228 (45) (n = 14)	115 (26) (n = 15)	168 (39) (n = 15)	-1.6 (6.1) (n = 11)
	*p*-value	0.71	0.008	<0.001	0.14	0.18
HIT n = 19	Pre	2.77 (0.55)	212 (48)	96 (29)	158 (42)	-0.7 (5.7) (n = 17)
	Post	3.08 (0.59)	239 (47)	111 (32)	178 (37)	1.7 (4.0) (n = 17)
	*p*-value	<0.001	<0.001	0.005	<0.001	0.05
Between group change Time x group	*p*-value	<0.001	<0.002	0.85	0.004	0.53

LIT, low intensity training group; HIT, High Intensity Training group. VO2max, Maximal oxygen uptake. Pmax, Maximum aerobic power. PLT1, Power at the first lactate threshold; PLT2, Power at the second lactate threshold; Sprinting fatigue ratio Ratio of (15 s sprint after durability test - 15 s sprint rest)/(15 s sprint rest).

There was no time x group difference [*p* = 0.91, ES = 0.33 (-0.44—1.16), CLES = 51%] in the sprinting fatigue ratio (Table 5). It increased in the HIT group, but no change was observed in the LIT group [LIT: n = 11, Δ sprinting fatigue ratio = +0.8 (8.6) p. p., *p* = 0.18, ES = 0.15 (-0.86—1.19), CLES = 73%; HIT: n = 17, Δ sprinting fatigue ratio = +2.4 (4.6) percentage points, *p* = 0.05, ES = 0.48 (0.06—0.98), CLES = 70%].

*Post-hoc correlations.* When both groups were studied together in terms of training induced changes between the drifts, VO_2max_, and power at LT_1_, the only noteworthy correlations were observed between Δ EE and Δ VE drift (r = 0.49, *p* = 0.003), and between Δ HR and Δ imputed LVET drift (r = -0.44, *p* = 0.008). The other correlations were small (|r| < 0.22, *p* > 0.22). Especially, change in VO_2max_ was not associated with change in any physiological drifts.

*Description of durability test.* RER did not change during 3 h durability test from 30 min to 180 min: from 0.93 (0.03) to 0.92 (0.02) during pre-test and from 0.92 (0.04) to 0.92 (0.02) during post-test (n = 35, both groups combined) with no differences between the groups. Body mass during the 3 h durability test was unchanged: +0.2 (0.6) kg in pre-test, and -0.1 (0.4) kg in post-test (n = 28, both groups combined) with no differences between the groups. Also, the water intake was similar in pre- and post-tests (*p* = 0.07, g = 0.59, groups combined). EE, VO_2_, Imputed LVET and SV during durability test are shown in Supplementary Digital Content 2.

## 4 Discussion

Our main finding was that there was no difference between improvement in durability after 10 weeks of both LIT and HIT based on averaged value consisted of reduced magnitude of the physiological drifts (-1.2 and -2.4 p. p. on average), postponed time of onset (25 and 29 min on average) and attenuated physiological strain. No adverse effects, i.e. increased drifts, shortened onset of drifts or amplified physiological strain, were detected at the group level in any individual variable.

### 4.1 Results compared to literature

Our EE drift (6%) was consistent with earlier studies. Recalculated from average values from tables and figures, VO_2_ drift between 4% and 7% has been reported during 90–180 min low-to moderate-intensity endurance exercise (Coggan et al., 1993; Carter et al., 2001; Rønnestad et al., 2011), but also values as high as 16% were reported (Hurley et al., 1986). In these studies, VO_2_ drift was reduced by endurance alone or concurrent endurance and strength training by 2–4 p. p., which is equal to our 3 p. p. decrease in EE drift. In these same studies, initial HR drift of 10%—20% was reduced by 3 – 9 p. p. in endurance only or concurrent training. In our study, HR drift was initially 14%. HR drift was reduced by 4 p. p. in the HIT group, while it was not changed after LIT. It should be noted, however, that training does not necessarily lead to enhanced durability. Concurrent training did not affect durability (physiological drifts) during a 1 h load carriage task (Wills et al., 2019), and endurance training in well-trained athletes did not lead to enhanced durability (Hausswirth et al., 2010; Rønnestad et al., 2011), nor did concurrent training (Hausswirth et al., 2010).

### 4.2 Durability and relative reduction in intensity

Durability can usually be increased by endurance training in recreationally active subjects (Hurley et al., 1986; Coggan et al., 1993; Phillips et al., 1996; Carter et al., 2001). However, as training simultaneously increases VO_2max_, the relative intensity during the prolonged tests in these studies were lower after the intervention. It is possible that after the intervention the first lactate threshold may have risen above tested intensity, thereby possibly moving the tested intensity to a different exercise zone. This provokes the question of whether durability itself is enhanced or the drifts are more restrained because of decreased relative intensity.

In our study, we assured that the intensity was in both test occasions initially at low intensity zone so that the origin of fatigue was similar at both times. Similar lactate concentrations in pre- and post-tests confirms the similar exercising zones. VO_2max_ increased only in the HIT group and the relative intensity was -6 p. p. lower (49% vs. 43% VO_2max_) in post-test compared to pre-test, while it remained unchanged in the LIT group. Hence, for the LIT group, durability was affected without increase in VO_2max_. This supports the idea that factors affecting VO_2max_ and durability may be different, or that durability can be affected both by improving VO_2max_ as well as by improving submaximal metabolism.

In a previous study (Carter et al., 2001), subjects cycled both the same relative and absolute intensities after a high-volume training intervention. Based on the study (Carter et al., 2001), one can deduce (after recalculations) that physiological drifts and strain are diminished by reduced relative intensity alone, and that increased durability (2.4 p. p. reduction in VO_2_ drift) might be possible when the same relative power is repeated. Further, concurrent endurance and heavy strength training has also shown that enhanced durability (reduced HR and VO_2_ drifts from recalculations) is possible with only a small simultaneous increase (+3%) in VO_2max_ (Rønnestad et al., 2011). Finally, power profile in a fatigued state changes in competitive cyclists during the competition season (Spragg et al., 2022), with only minor changes in maximum exercise performance.

### 4.3 Decreased physiological strain

Formerly, the RPE response has been reduced after training during prolonged exercise (Rønnestad et al., 2011; Wills et al., 2019), which is consistent with the present findings. We observed that there was a decrease in submaximal exercise HR, which usually is reported to stem from increased plasma and total blood volume (Green et al., 1990). Structural or functional adaptations within the myocardium take longer and have not been found after a few weeks of endurance training (Bonne et al., 2014). Acute plasma volume expansion decreases HR considerably, up to 10–15 bpm (Green et al., 1990). However, it does not alter, and hence explain changes in, HR drift (Green et al., 1990; Grant et al., 1997).

In creased HRV indices may also be a consequence of plasma expansion (Frank et al., 2001). Further, catecholamine responses to regular exercise in a stable trained state are reduced (Phillips et al., 1996; Carter et al., 2001). Hence, reduced sympathetic effect on the heart may lead to increased HRV, which indicates positive changes in balance of the autonomic nervous system.

### 4.4 Underlying mechanistic reasons to drifts

The six physiological drifts were investigated to gain a wide physiological drift profile. EE represents the overall physiological strain of exercise, and RPE represents perceived strain. HR is the most utilized marker of relative intensity during prolonged exercise, and with SV, they completely represent the circulating blood flow. LVET illustrates the sympathetic activity of the heart. VE, in addition to being strongly linked to CO_2_ output, also portrays complex exercise hyperpnea.

EE drift is related to depletion of glycogen stores in slow-twitch muscle fibers of agonist muscles, after which the body needs to recruit less efficient co-agonists and fast-twitch fibers to maintain the constant power output (Krustrup et al., 2004). Also elevated O_2_ cost of fatigued muscle (Hopker et al., 2017) is nominated to cause EE drift. Thus, it is presumable that at least one of these processes was slowed during our intervention. Glycogen content of a muscle is increased by endurance training (Murray and Rosenbloom, 2018), as well as fat oxidation capacity and efficiency (Phillips et al., 1996). Hence, after the training, the need to activate less efficient and highly fatigable fast-twitch fibers is postponed, leading to reduced EE drift. Additionally, endurance training alters type II fibers by augmenting fatigue-resistance to act more like the type I fibers (Seene et al., 2007), so their mechanical efficiency might not drop so considerably during prolonged exercise. As endurance training also enhances mitochondrial mass and function (Bishop et al., 2014), energy production can be spread through larger mass, inflicting smaller oxidative damage during prolonged cycling so that O_2_ -cost is not elevated as greatly.

Due to the exercise intervention of the present study, HR drift was reduced in the HIT group. An increased sympathetic activation, core temperature, and fatigue of the neuromuscular system have been reported to be the reasons for HR drift (Coyle and González-Alonso, 2001). Core temperature was not measured in our study, and neuromuscular fatigue was negligible, as measured with 15 s maximum sprint. While HRV is a marker for autonomic nervous system balance, LVET and pre-ejection period can indicate chronotropic sympathetic activity (Thayer and Uijtdehaage, 2001). As the LVET drift was reduced in the HIT group in trained state, and its onset was postponed, it is possible that dampened sympathetic activity during durability tests may play a role in reducing HR drift. This observation is supported by the positive correlation between change in HR and LVET drifts. Increased HRV responses may further support our interpretation.

Durability can be seen as a form of fatigue resistance. However, the term ‘fatigue resistance’ has evolved greatly to different directions during its history. Its interpretations include, for example, an ability to resist fall in potentiated twitch force (Ducrocq et al., 2021) or in force during electrical stimulation (Morris et al., 2008), time to exhaustion (Lindsay et al., 1996), and a fraction of VO_2max_ used during a time trial (Hawley et al., 1997). Fatigue resistance is usually connected to neuromuscular fatigue during maximal and short performances. Durability, in turn, is a specifically defined way to study fatigue resistance associated to prolonged and submaximal performances using physiological variables.

### 4.5 Training induced changes

The groups had similar baseline physical activity habits consisting in mostly low intensity activity. Training intervention induced changes in measured responses in our study in both groups pointed toward improved durability, indicating improved durability with the exception of imputed LVET in the LIT group. Based on effect sizes, LIT and HIT did not seemed to increase drifts or their onset in large amount, despite that the subjects had not undertaken systematic endurance training and a majority of them had not engaged in 3 h exercise before. This is quite a contrast to other endurance capacity markers such as VO_2max_ (Wen et al., 2019) and time trial ability (Rosenblat et al., 2021), which are usually quite distinctly enhanced in untrained subjects. Indeed, VO_2max_ was also distinctly elevated in our HIT group with very large effect size.

Keeping power below LT_1_ in the LIT group resulted in a mean HR of 63% HR_max_. The sufficient exercise intensity to improve endurance capacity is at least 3 metabolic equivalents (Bull et al., 2020) which, in the case of the LIT group, corresponds to 58 (9)% HR_max_ in pre-test. As training did not induce changes in VO_2max_ in the LIT group, it is possible that the intensity was not high enough for these subjects (Swain and Franklin, 2002; Laursen, 2010), which leads to a conclusion that intensity to improve VO_2max_ could be higher than the intensity required for durability improvements.

It was surprising that high-volume LIT, which mimics very closely the durability test and challenges the same origins of fatigue (Black et al., 2017; Burnley and Jones, 2018), did not induce larger increases in durability. Earlier, 65% VO_2max_-intensity training produced quite clear changes in the VO_2_ drifts (Phillips et al., 1996; Carter et al., 2001), suggesting that durability may also be intensity dependent and one cannot simply compensate lower intensity with longer exercise bouts. Other reason might be that although the origins of fatigue are different in low and high intensity zones, high intensity zone could nevertheless stimulate similarly central neuromuscular function as can be deduced by similar M-wave decrease after low and high intensity exercise (Black et al., 2017) and they both deplete glycogen storages (Gollnick et al., 1974). Hence, they both could challenge similar abilities to resist fatigue at durability test. In our durability test constant energy intake was present, which prevented the influence of the possible enhanced fat oxidation capacity.

### 4.6 Limitations of our study

It seems that test-retest reliability measures have not been undertaken, although multiple studies have been conducted on physiological drifts in prolonged submaximal exercise (Ekelund, 1967; Phillips et al., 1996; Carter et al., 2001; Coyle and González-Alonso, 2001; Rønnestad et al., 2011; Mullins et al., 2015; Wills et al., 2019; Smyth et al., 2022). Hence, it is not known how sensitive physiological drifts are to changes in environment and preparation.

Nutrition was not controlled during the training, although the relevance of carbohydrate ingestion was stressed to participants during the training. Ingesting carbohydrates during the durability test reduced the importance of nutrition status, but it does not eliminate it completely (Tsintzas et al., 2001).

Change in plasma volume during 3 h durability test was not monitored. However, the body mass was not reduced, and the plasma volume reduction in prolonged low intensity exercise at lower than 50% VO_2max_ intensity is minimal (Grant et al., 1997). Therefore, it can be deduced that acute plasma volume change during 3 h test had minimal effect to the outcomes.

There are sex differences in physiological responses to exercise (e.g. fat oxidation) (Purdom et al., 2018). As such, it might be that there are sex-specific optimal training protocols to improve durability. Our sample size was not planned to assess possible sex differences in the studied variables. Menstrual cycle was not controlled, but ingesting carbohydrates during the durability test minimized its effect (Campbell et al., 2001).

Our sample size (n = 35) was quite favorable as compared to previous intervention studies (n = 8–20) (Coggan et al., 1993; Phillips et al., 1996; Carter et al., 2001; Frank et al., 2001; Hausswirth et al., 2010; Rønnestad et al., 2011; Bonne et al., 2014; Wills et al., 2019). Nevertheless, a higher number of subjects may have enabled us to observe differences between the study groups and/or training protocols.

### 4.7 Future directions

The existing research on durability has mostly been reported by observational studies using existing training databases (Van Erp et al., 2021; Smyth et al., 2022; Spragg et al., 2022). Although that allows one to reach a high number of subjects, carefully conducted laboratory studies are needed to deepen the understanding of durability, its links to other possible physiological sources (e.g. endocrinal, body temperature, and muscle activity patterns drifts), and adaptability to training.

## 5 Conclusion

Our study was one of the first interventions on focusing durability, and it showed that 10-week high- and low-intensity training programs increased durability among untrained subjects, and no differences between the training groups were noticed. Increased VO_2max_ facilitated 3 h cycling only in the high-intensity training group, although the change in VO_2max_ did not correlate with the change in durability. Hence, it seems that durability and VO_2max_ are not strongly related with each other and are driven by different physiological mechanisms. Despite durability enhanced among untrained people, a 10-week intervention did not alter drifts and their onsets in a large amount, even though it attenuated physiological strain.

## Data Availability

The raw data of this article is not yet available due to the agreement with the collaborative company and further papers will also be written from the same data. However, the corresponding author is willing to give further information in this regard.
