# Supplementary material for: Durability is improved by both low and high intensity endurance training
Source: Front Physiol. 2023 Feb 16;14:1128111. doi: 10.3389/fphys.2023.1128111 (PMC9977827; doi:10.3389/fphys.2023.1128111)
Supplement: Supplementary file 1 [file DataSheet2.pdf]

Supplementary Material 2: Description of 3 h test values

Durability is improved by both low and high intensity endurance training

Pekka Matomäki*, Olli J. Heinonen, Ari Nummela, Jari Laukkanen, Eero-Pekka Auvinen, Leena Pirkola, Heikki Kyröläinen

*** Correspondence:** Corresponding Author: pmatomaki@gmail.com

Supplementary 2, Table 1. Physiological responses during 3 h durability test (mean, SD), and p-value for the pre- vs. post-test difference for the LIT group.

			Rest	30 min	60 min	90 min	120 min	150 min	180 min
LIT (n = 16)	Power	pre		86 (24) W and 46 (4)% VO_{2max}					
		post		86 (24) W and 46 (4)% VO_{2max} (p = 1.0)					
	EE (kJ / min)	pre	6.6 (1.2)	28.6 (6.0)	29.6 (6.3)	29.7 (6.5)	29.9 (6.6)	30.0 (6.7)	30.5 (7.2)
		post	6.5 (1.2) (n=15)	28.7 (5.7)	29.6 (6.3)	29.3 (6.3)	29.6 (6.4)	30.0 (6.7)	30.0 (6.9)
		p-value	0.59	0.64	0.98	0.26	0.52	0.33	0.23
	VO_2 (ml /min)	pre	318 (57)	1372 (284)	1422 (298)	1425 (310)	1439 (314)	1444 (318)	1473 (347)
		post	318 (54)	1384 (268)	1424 (299)	1405 (301)	1425 (305)	1446 (321)	1448 (330)
		p-value	0.48	0.45	0.90	0.28	0.51	0.94	0.25
	Imputed LVET (ms)	pre (n = 13)	341 (57)	273 (40)	268 (35)	266 (33)	265 (30)	266 (32)	263 (31)
		post (n = 13)	341 (63)	282 (44)	277 (32)	277 (35)	272 (29)	273 (36)	268 (29)
		p-value	1.0	0.18	0.07	0.04	0.13	0.15	0.04
	Imputed SV (ml)	pre (n = 13)	97 (18)	111 (24)	107 (24)	106 (24)	106 (21)	104 (21)	103 (20)
		post (n = 13)	99 (15)	113 (21)	110 (20)	109 (19)	107 (19)	106 (17)	107 (18)
		p-value	0.68	0.77	0.56	0.60	0.77	0.53	0.30

LIT Low-intensity training group. *EE* Energy expenditure. *VO_2* oxygen uptake. *LVET* Left ventricular ejection time. *SV* Stroke volume.

Supplementary 2, Table 2. Physiological responses during 3 h durability test (mean, SD), and p-value for the pre- vs. post-test difference for the HIT group.

			Rest	30 min	60 min	90 min	120 min	150 min	180 min
HIT (n = 19)	Power	pre		82 (22) W and 49 (4)% VO_{2max}					
		post		82 (22) W and 43 (5)% VO_{2max} ($p < 0.001$)					
	EE (kJ / min)	pre	6.0 (1.1)	28.4 (6.3)	29.0 (6.4)	29.3 (6.8)	29.6 (7.0)	29.6 (7.3)	30.3 (7.5)
		post	6.1 (1.4)	27.5 (6.1)	28.0 (6.2)	27.9 (6.0)	28.2 (6.5)	28.3 (6.2)	28.3 (6.3)
		p-value	0.65	0.25	0.11	0.05	0.04	0.05	0.003
	VO_2 (ml / min)	pre	293 (55)	1365 (305)	1394 (310)	1403 (328)	1424 (336)	1420 (352)	1458 (357)
		post	297 (65)	1328 (290)	1350 (296)	1336 (287)	1353 (308)	1359 (294)	1362 (298)
		p-value	0.63	0.28	0.12	0.06	0.04	0.06	0.002
	Imputed LVET (ms)	pre (n =16)	367 (43)	283 (20)	282 (23)	278 (22)	276 (16)	270 (18)	268 (22)
		post (n = 16)	360 (43)	288 (28)	290 (26)	288 (26)	284 (25)	285 (27)	279 (24)
		p-value	0.47	0.40	0.12	0.03	0.03	0.01	0.06
	Imputed SV (ml)	pre (n =16)	98 (14)	114 (18)	115 (19)	113 (17)	112 (20)	112 (19)	112 (19)
		post (n = 16)	100 (18)	120 (28)	119 (27)	118 (27)	117 (28)	117 (27)	117 (30)
		p-value	0.63	0.22	0.37	0.26	0.24	0.24	0.28

HIT High-intensity training group. *EE* Energy expenditure. *VO_2* oxygen uptake. *LVET* Left ventricular ejection time. *SV* Stroke volume.
