# Supplementary material for: Durability is improved by both low and high intensity endurance training
Source: Front Physiol. 2023 Feb 16;14:1128111. doi: 10.3389/fphys.2023.1128111 (PMC9977827; doi:10.3389/fphys.2023.1128111)
Supplement: Supplementary file 2 [file DataSheet1.pdf]

Supplementary Material 1: Training details

Durability is improved by both low and high intensity endurance training

Pekka Matomäki*, Olli J. Heinonen, Ari Nummela, Jari Laukkanen, Eero-Pekka Auvinen, Leena Pirkola, Heikki Kyröläinen

*** Correspondence:** Corresponding Author: pmatomaki@gmail.com

1 Training details

There were two groups: low intensity training (LIT) and high intensity training (HIT) groups for which the subjects were randomly divided. Both groups had 10 training weeks. During week 6, a follow-up VO_{2max} test was done to check training intensities, and weeks 3 and 7 were load reduction weeks for enhancing recovery.

There were pre-scheduled, 22-line progression table for training for LIT (Supplementary Table 3) and HIT (Table 4) groups with equal calculated training loads, in an ascending order (see Supplementary Figure 1). Each subject started with progression line 1. After each training week (excluding load reduction weeks), subjects were asked 'How much the training has strained your week in the scale 0–10?', and 10-scale RPE-table were used. They then moved up the progression table depending on the strain of the preceding week following Supplementary Table 1.

Supplementary 1, Table 1. Weekly RPE (rate of perceived effort) and its influence on progression.

Weekly RPE	Progression
0 – 2	+ 4
3 – 4	+ 3
5 – 6	+ 2
7 – 8	+ 1
9 – 10	0 (the same week was repeated)

Training was monitored by distributing cycling power output to five zones, and for each zone a weighting factor was linked (Cejuela-Anta and Esteve-Lanao, 2011) (Supplementary Table 2). Training load was calculated multiplying the factor by the time spend in zone.

Supplementary 1, Table 2. Definitions of training zones and weighting factors associated to them.

Zone	Definition	Weighting factor
Z1	below LT_1 – 10 W	1
Z2	between LT_1 – 10 W to LT_1 + 10 W	2
Z3	between LT_1 + 10 W to LT_2 – 10 W	3
Z4	between LT_2 – 10 W to LT_2 + 10 W	4
Z5	above LT_2 + 10 W	7.5

LT_1 First lactate threshold. LT_2 Second lactate threshold.

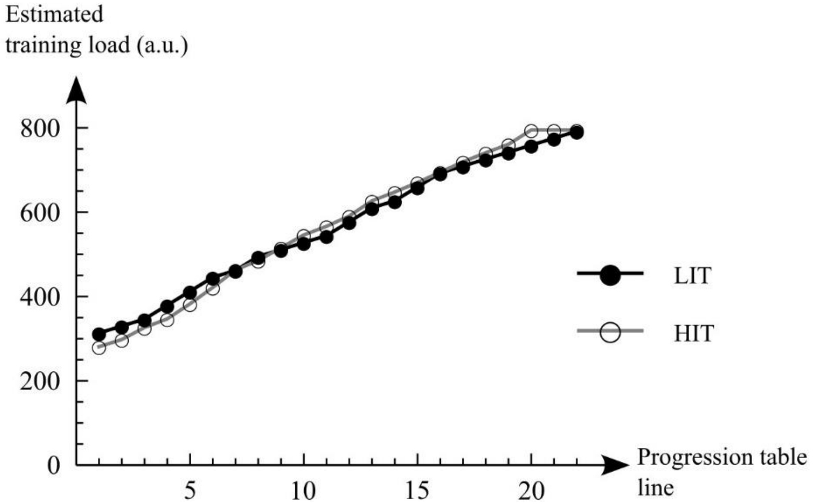

Supplementary 1, Figure 1. Estimated training loads of the progression tables for training of LIT and HIT groups. *LIT* Low intensity training. *HIT* high intensity training. *a.u.* arbitrary unit

The subjects were encouraged to consume enough carbohydrates during the intervention.

1.1 Equalizing training

Although training scheduling table was equalized by training load calculation, the realization of training loads were 88 (14) in LIT and 55 (8) in HIT ($p < 0.001$, $g = 3.0$). There is no clear consensus how the training loads between LIT and HIT should be equalized (Normand-Gravier et al., 2022; Passfield et al., 2022). We used individualized training load progression, where the training load was equalized individually so that subjects would have similar training load relative to their maximum tolerable level. All in all, subjects in the LIT group felt training easier and thus progressed at faster pace than subjects in the HIT group.

1.2 Training in LIT group

In Supplementary Table 3 below 22-line scheduled progression table for LIT group is given. After the first training week (which was the first line in the progression table) the progression was individualized moving forward 0–4 lines based on the weekly RPE (Supplementary Table 1). Five (out from 16) subjects progressed to the last progression line 22.

LIT group trained outdoors with their own bikes. A possibility for indoors cycling with trainer or Wattbike Trainer (Wattbike Ltd., Nottingham, UK) were given. Three (out from 16) subjects did their training completely indoors, and the others did 3 % of their training indoors.

Training power in LIT group was solely below first lactate threshold (LT_1), and the training power was monitored and feedback given weekly. Especially, if steep hills were not possible to cycle below LT_1 -power, subjects were adviced to walk up the hills.

During training week 6, a follow-up VO_{2max} -test was done, which replaced a “moderately long” (1 – 1,75 h) training ride.

Supplementary 1, Table 3. Scheduled training progression table for LIT group. Training power was below first lactate threshold. Subjects were free to do the exercise of the week at the order they preferred.

	Type of training					
Progression line	Long exercise	Moderately long exercise	Short exercise	Amount of exercises (/ week)	Cumulative training time (h)	Estimated training load (a.u.)
1	1 x 1.5 h	1 x 1 h	3 x 45 min	5	4.75	313.5
2	1 x 1.5 h	2 x 1 h	2 x 45 min	5	5	330
3	1 x 1.75 h	2 x 1 h	2 x 45 min	5	5.25	346.5
4	1 x 2 h	1 x 1.25 h;<br>1 x 1 h	2 x 45 min	5	5.75	379.5
5	1 x 2.5 h	1 x 1.25 h<br>1 x 1 h	2 x 45 min	5	6.25	412.5
6	1 x 2.5 h	1 x 1.75 h<br>1 x 1 h	2 x 45 min	5	6.75	445.5
7	1 x 2.5 h;<br>1 x 2 h	1 x 1 h	2 x 45 min	5	7	462
8	1 x 3 h;<br>1 x 2 h	1 x 1 h	2 x 45 min	5	7.5	495
9	1 x 3 h<br>1 x 2 h	2 x 1 h	1 x 45 min	5	7.75	511.5
10	1 x 3 h;<br>1 x 1.5 h	2 x 1 h	2 x 45 min	6	8	528
11	1 x 3 h;<br>1 x 2 h;	1 x 1 h	3 x 45 min	6	8.25	544.5
12	1 x 3 h;<br>1 x 2.5 h;	1 x 1 h	3 x 45 min	6	8.75	577.5
13	1 x 3 h;<br>1 x 2.5 h	1 x 1.25 h<br>1 x 1 h	2 x 45 min	6	9.25	610.5
14	1 x 3 h;<br>1 x 2.5 h	2 x 1.25 h	2 x 45 min	6	9.5	627
15	2 x 3 h	2 x 1.25 h	2 x 45 min	6	10	660
16	1 x 3.5 h<br>1 x 2.5 h	2 x 1.5 h	2 x 45 min	6	10.5	693
17	1 x 3.5 h<br>1 x 3 h	1 x 1.5 h<br>1 x 1.25 h	2 x 45 min	6	10.75	709.5
18	2 x 3 h	2 x 1 h<br>2 x 1.5 h		6	11	726
19	1 x 3.5 h<br>2 x 2 h	1 x 1.5 h<br>1 x 1.25 h<br>1 x 1 h		6	11.25	742.5
20	1 x 3.5 h<br>3 x 2 h	2 x 1 h		6	11.5	759
21	1 x 4 h<br>1 x 3 h	2 x 1.5 h<br>1 x 1 h	1 x 45 min	6	11.75	775.5
22	1 x 4 h<br>1 x 3.5 h	3 x 1 h<br>1 x 1.5 h		6	12	792
Load reduction week	1 x 1.5 h		2 x 45 min	3	3	198

LIT Low intensity training. *HIT* high intensity training. *a.u.* arbitrary unit

1.3 Training in HIT group

In Supplementary Table 4 below 22-line scheduled progression table for HIT group is given. After the first training week (which was the first line in the progression table) the progression was individualized moving forward 0–4 lines based on the weekly RPE (Supplementary Table 1). Five (out from 16) subjects progressed to the last progression line 22. Eight (out from 19) subjects progressed to at least progression table line 15, and none further than 19.

HIT group trained indoors with Wattbike Trainer (Wattbike Ltd., Nottingham, UK) or trainer with their own bicycles with Rally RK200 dual-sensing power meters. Each session started with 10 minutes warm up (< 60 W) after which the prescribed intervals were made. The exercise ended in 10 minutes cool down (<60 W).

In the interval section, the work intensity was initially 110 % of second threshold power (LT_2) ($\pm 15\ W$), and recovery intensity was < 60 W. The length of recovery interval was ¾ of the work interval. The training power, heart rate, and RPE were monitored, and feedback given weekly. If all training during the week had RPE ≤ 6 and HR did not rise above LT_2 -threshold, then the power of work intervals was increased by 10 %. In the lines 15–22 of the scheduled training progression table, the intensity of the work interval was "maximal sustainable effort".

During training week 6, a follow-up VO_{2max} -test was done, which replaced the interval exercise from that week with the lowest training load.

Supplementary 1 Table 4. Scheduled training progression table for HIT group. Power during the work intervals were initially 110 % LT_2. Warmup, Cool-down, and recovery periods were done below 60 W. In the exercises marked in red, the work intensity was maximal sustainable effort. Subjects were free to do the exercise at the order they preferred. It was recommended to time the exercises evenly to the week with at least 48 h between two sessions.

	Type of training					
Progression line	Exercise type 1	Exercise type 2	Amount of exercises (/ week)	Cumulative HIT time (min / week)	Cumulative LIT time (min / week)	Estimated training load (a.u.)
1	2 exercises:<br>5 x 3 min @ HIT		2	30	56	281
2	2 exercises:<br>4 x 4 min @ HIT		2	32	58	298
3	2 exercises:<br>5 x 3.5 min @ HIT		2	35	64	326.5
4	1 exercise:<br>3 x 6 min @ HIT	1 exercise:<br>5 x 4 min@ HIT	2	38	62	347
5	1 exercise:<br>5 x 4 min @ HIT	1 exercise:<br>5 x 4.5 min @ HIT	2	42.5	64	382.75
6	3 exercises:<br>3 x 5 min @ HIT		3	45	84	421.5
7	1 exercise:<br>5 x 4 min @ HIT	2 exercises:<br>3 x 5 min @ HIT	3	50	88	463
8	1 exercise:<br>5 x 4 min @ HIT	2 exercises:<br>3 x 5.5 min @ HIT	3	53	88	485.5

9	2 exercises:<br>5 x 4 min @ HIT	1 exercise:<br>3 x 5.5 min @ HIT	3	56.5	92	515.75
10	3 exercises:<br>4 x 5 min @ HIT		3	60	96	546
11	1 exercise:<br>3 x 7 min @ HIT	2 exercises:<br>7 x 3 min @ HIT	3	63	94	566.5
12	3 exercises:<br>4 x 5.5 min @ HIT		3	66	96	591
13	2 exercises:<br>6 x 4 min @ HIT	1 exercise:<br>4 x 5.5 min @ HIT	3	70	102	627
14	2 exercises:<br>5 x 5 min @ HIT	1 exercise:<br>5 x 4.5 min @ HIT	3	72.5	104	647.75
15	3 exercises:<br>5 x 5 min @ HIT		3	75	108	670.5
16	1 exercise:<br>7 x 4 min @ HIT	2 exercises:<br>5 x 5 min @ HIT	3	78	110	695
17	2 exercises:<br>7 x 4 min @ HIT	1 exercise:<br>5 x 5 min @ HIT	3	81	112	719.5
18	1 exercise:<br>4 x 7 min @ HIT	2 exercises:<br>7 x 4 min @ HIT	3	84	111	741
19	1 exercise:<br>6 x 5 min @ HIT	2 exercises:<br>7 x 4 min @ HIT	3	86	116	761
20	3 exercises:<br>6 x 5 min @ HIT		3	90	120	795
21	3 exercises:<br>5 x 6 min @ HIT		3	90	120	795
22	3 exercises:<br>5 x 6 min @ HIT		3	90	120	795
Load reduction week	2 exercises:<br>3 x 3 min @ HIT		2	18	38	183

LIT Low intensity training. *HIT* high intensity training. *a.u.* arbitrary unit

1.4 References

Cejuela-Anta, R., and Esteve-Lanao, J. (2011). Training load quantification in triathlon. *Journal of Human Sport and Exercise* 6, 218–232.

Normand-Gravier, T., Britto, F., Launay, T., Renfree, A., Toussaint, J. F., and Desgorces, F. D. (2022). Exercise Dose Equalization in High-Intensity Interval Training: A Scoping Review. *Int J Environ Res Public Health* 19. doi: 10.3390/ijerph19094980.

Passfield, L., Murias, J. M., Sacchetti, M., and Nicolo, A. (2022). Validity of the Training-Load Concept. *Int J Sports Physiol Perform* 17, 507–514. doi: 10.1123/ijspp.2021-0536.
